# How Load-Carrying Ants Avoid Falling Over: Mechanical Stability during Foraging in *Atta vollenweideri* Grass-Cutting Ants

**DOI:** 10.1371/journal.pone.0052816

**Published:** 2013-01-02

**Authors:** Karin Moll, Flavio Roces, Walter Federle

**Affiliations:** 1 Department of Zoology, University of Cambridge, Cambridge, United Kingdom; 2 University College Freiburg, University of Freiburg, Freiburg, Germany; 3 Department of Behavioural Physiology and Sociobiology, Biocenter, University of Würzburg, Würzburg, Germany; University of Zurich, Switzerland

## Abstract

**Background:**

Foraging workers of grass-cutting ants (*Atta vollenweideri*) regularly carry grass fragments larger than their own body. Fragment length has been shown to influence the ants’ running speed and thereby the colony’s food intake rate. We investigated whether and how grass-cutting ants maintain stability when carrying fragments of two different lengths but identical mass.

**Principal Findings:**

Ants carried all fragments in an upright, backwards-tilted position, but held long fragments more vertically than short ones. All carrying ants used an alternating tripod gait, where mechanical stability was increased by overlapping stance phases of consecutive steps. The overlap was greatest for ants carrying long fragments, resulting in more legs contacting the ground simultaneously. For all ants, the projection of the total centre of mass (ant and fragment) was often outside the supporting tripod, i.e. the three feet that would be in stance for a non-overlapping tripod gait. Stability was only achieved through additional legs in ground contact. Tripod stability (quantified as the minimum distance of the centre of mass to the edge of the supporting tripod) was significantly smaller for ants with long fragments. Here, tripod stability was lowest at the beginning of each step, when the center of mass was near the posterior margin of the supporting tripod. By contrast, tripod stability was lowest at the end of each step for ants carrying short fragments. Consistently, ants with long fragments mainly fell backwards, whereas ants carrying short fragments mainly fell forwards or to the side. Assuming that transporting ants adjust neither the fragment angle nor the gait, they would be less stable and more likely to fall over.

**Conclusions:**

In grass-cutting ants, the need to maintain static stability when carrying long grass fragments has led to multiple kinematic adjustments at the expense of a reduced material transport rate.

## Introduction

The fitness of an animal primarily depends on the quality and quantity of food [1]. In social insects, individual workers are responsible for the nutrient gain of the entire colony and therefore its survival. Hence, the behavior of foraging workers is considered to be shaped by natural selection to maximize the colony’s energy intake [2]. Since most social insects are central-place foragers, the transport of food items back to the nest represents an important part of the energetic cost of foraging. The effects of load size on locomotory performance are therefore essential for the understanding of foraging economics. The influence of load mass on locomotion and transport rates has been studied in many social insects (e.g. *Veromessor pergandei* [3], *Atta colombica* [4], *Atta cephalotes* [5], [6], *Dorymyrmex goetschi* [7], *Apis mellifera* [8], [9]). However, when insects carry large objects with their mandibles, the load is located away from the body centre, resulting in a significant shift of the total centre of mass. Hence, in addition to the direct effects of the load mass on carrying speed, large off-centre loads may increase the insects’ risk of falling over. Such stability constraints during the transport of loads, as previously suggested by Zollikofer [10], have not been investigated, although stability during locomotion represents a fundamental problem for pedestrian animals [11].

In order to maintain static stability during slow terrestrial locomotion, the projection of the centre of mass needs to lie within the polygon of support, otherwise the animal will fall (unless it can cling to the ground) [11]. Unlike humans and four-legged vertebrates, insects mostly seem to maintain static stability during locomotion. The minimum requirement for static stability is a supporting tripod; many insects move with an alternating tripod gait, with the front and hind legs of one side moving in synchrony with the mid leg of the opposite side [12]. A shift of the centre of mass has been shown to strongly influence running kinematics in ants [10]. Off-centre loads may require more legs in ground contact at any given time to provide sufficient stability, likely limiting the insects’ running speed and thus the rate at which material is transported. However, quantitative measurements of static stability during load transport are missing and it is unclear to what extent foraging economics are influenced by stability constraints.

Leaf-cutting ants provide an excellent system to investigate the effects of stability constraints on foraging, since individual ants carry fragments of leaves larger than their own bodies [5], [13], [14]. Stability constraints are particularly obvious in leaf-cutting ants that harvest grass, the so-called grass-cutting ants [15], [16], [17]. These ants carry fragments of grass blades by holding them with their mandibles in an upright position that is slightly tilted backwards. In our study species *Atta vollenweideri*, the lengths of grass fragments cut in the field range from approximately 5 to 60 mm [18] and can consequently exceed by many times the ants’ body length (body length with extended gaster of foraging ants in our laboratory colony: 5.0–7.6 mm).

Grass-cutting ants reduce a backward shift of the centre of mass during grass transport by holding long fragments more steeply than shorter ones [19]. Nevertheless, the transport of long fragments is still associated with a backward shift of the centre of mass. Running speed in grass-cutting ants decreases with increasing fragment length, independent of fragment mass, leading to a reduced rate at which material is transported to the nest [20].

In this study we investigated the effect of fragment length on static stability in grass-cutting ants by quantifying stability margins in ants that carried artificial paper fragments of different length, but the same mass. To investigate the influence of the ants’ gait pattern and fragment angle (i.e. the steepness of the fragments) on static stability, we calculated stability margins for different assumed gaits and fragment angles. We further tested to what extent the ants’ physiological ability to move their heads up and down enables a substantial adjustment of the angles at which fragments could be transported.

## Materials and Methods

### Study Animals

To quantify stability during the transport of grass fragments, a large laboratory colony of *Atta vollenweideri* was used. Ants were kept at a 12∶12 day-night cycle at approximately 25°C and 40–50% humidity. Before experimental days ants were fed with an *ad libitum* supply of dog rose leaves (*Rosa canina*) and honey water. To minimize the effect of body size, only intermediate sized ants between 3.5 and 5.5 mg were analyzed.

### Tested Fragments

The effect of fragment length on running stability was investigated using standardized paper fragments of two different lengths (15 and 30 mm), henceforth called “short” and “long”. Paper of two different thicknesses (80 and 160 g m^−2^) was used to keep the width (2 mm) and the mass (short: 5.03±0.18 mg, long: 4.96±0.15 mg) of the fragments constant. Fragment length was chosen based on field measurements of naturally carried grass fragments by *A. vollenweideri* [18]. 30 mm is within the length range (approximately 5–35 mm) of fragments carried by workers of 3.5–5.5 mg body mass. To make the fragments sufficiently attractive to the ants, they were soaked in orange juice for at least 1 h and then dried.

### Experimental Setup and Procedure

**Figure 1:**
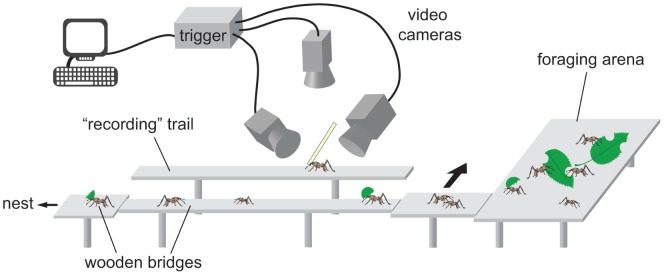
Experimental setup to video record load-carrying ants. Short and long paper fragments were presented on a foraging arena at a distance of 4.5 m to the nest. In order to keep the ants foraging, they could access dog rose leaves in the foraging arena. Using a moveable wooden bridge, ants that carried a paper fragments were led individually to the recording trail, where they were filmed with three synchronized video cameras at 50 fps.

Experiments were carried out as described in Moll et *al.* [19]. Long and short fragments were presented one after the other (in randomized order) on a foraging arena at a running distance of 4.5 m to the nest, which was always accessible to the ants via wooden bridges (Fig. 1). In order to keep the ants foraging, dog rose leaves were provided in the foraging arena. Paraffin oil on the underside of the bridges prevented the ants from leaving the trail. Using a moveable bridge, homebound ants that carried a paper fragment were led individually onto a separate “recording” trail (width: 2 cm). To encourage voluntary and stereotyped running on the “recording” trail, ants were fed with small amounts of dog rose leaves for at least 1 h prior to the experiments and foraging ants were led across this trail to establish a sufficient pheromone trail. This procedure was repeated at least every 2 h or earlier when the ants started to stray off their course to the nest. Ants were collected with forceps at the end of the recording trail. Individual ants were filmed with three synchronized high-speed cameras (A602f, Basler Vision Technologies, Ahrensburg, Germany) at 50 fps on the “recording” trail (Fig. 1). One camera filmed the ant from the top to record the ant’s footfall positions on the surface and body coordinates. Two cameras filmed the ant laterally to record the three-dimensional position of the fragment. The lateral views were also used to reconstruct footfall positions and body coordinates in case they were obstructed by the fragment in the top view recording. Recorded ants were collected at the end of the trail and ant and fragment were weighed separately to the nearest 0.01 mg using a microbalance (MC5, Sartorius, Göttingen, Germany).

In the top view recordings, the distal end of the tarsi, the mandibles’ tip and the posterior end of the thorax were digitized in every frame over a sequence of five step cycles. Trials in which the ants held on to the edges of the trail bridge were discarded. Pixel distances were converted to lengths after calibration with a known distance (0.080 mm/pixel).

To characterize the ants’ gait, we identified for each leg the timing of swing (protraction) and stance (retraction) phase. Using the start of the swing phase for the left front leg as a reference, we calculated the mean phase for each of the other five legs as the time difference between the reference leg and the given leg, divided by the period (sum of protraction and retraction time) of the reference leg. This calculation leads to a dimensionless number ranging from −1 to +1 (negative values for legs lifted off the ground before the reference leg, positive values for legs lifted after the reference leg) [21].

To compare phases between ants that carried short and long fragments the mean delay in phase of legs of left and right tripods were determined.

To measure the angle of the fragment relative to the surface and the position of the fragment’s centre of mass (*CoM*_f_), two points along the fragment, the fragment’s centre of mass and three points on the surface were digitized in both lateral recordings over a sequence of three step cycles. These points were reconstructed three-dimensionally using the Direct linear transformation (DLT) method according to Abdel-Aziz and Karara [22]. For camera calibration, 12 non-coplanar points forming a cuboid of known dimensions (obtained by moving a 10×5 millimetre grid visible for all cameras over a distance of 10 mm through the field of view using a MM-3 micromanipulator (0.01 mm resolution; Narishige International, London) were recorded in all cameras and digitized. The points’ dimensions and the camera coordinates were used to calculate 11 DLT parameters. These coefficients allowed us to calculate three-dimensional coordinates of points digitized in at least two cameras. The angle of the fragment relative to the surface was calculated vectorially. The cameras’ DLT coefficients allowed us to project the position of *CoM*_f_ into the two-dimensional top view recording.

For any tested ant, the anterior-posterior position of the ant’s centre of mass *dCoM*_a_ (distance from the tip of the mandibles in mm) was estimated based on the allometric equation *dCoM*_a_ = 1.69*M_a_* ^0.28^, where *M_a_* represents the ant’s body mass (in mg; derived from unpublished measurements in *A. vollenweideri*). The two-dimensional position of the ant’s centre of mass *CoM*_a_ was calculated with the help of a vector along the ant’s body axis (from the mandible’s tip to the posterior end of the thorax). From *CoM*_f,_ *CoM*_a_ and the ant’s and fragment’s mass *M_a_* and *M_f_*, we calculated for every frame the two-dimensional (projected) position of the total centre of mass *CoM*_t_ (ant+fragment).

### Stability Margin

Static stability is achieved when the total centre of mass lies within the polygon of supporting legs. The “stability margin”, which is defined as the shortest distance between the total centre of mass and the margin of the polygon of support [11], was measured to the nearest 0.01 mm for every frame. Positive values indicate that *CoM*_t_ is located within the polygon of support, whereas negative values indicate that *CoM*_t_ is outside.

To quantify stability during the transport of short and long fragments, we used not only the polygon of support as a reference but also an idealized, reduced version of the gait, with non-overlapping alternating tripods (i.e. exactly three legs supporting the body at any time). When less than six legs were in ground contact, we defined the “supporting tripod” as the only tripod with all three legs in stance. In situations where both tripods were in stance simultaneously, the tripod with the larger stability margin was defined as the tripod of support. To control for the size of the tripod, the maximum possible stability margin (i.e. the radius of the tripod’s incircle) was measured for every frame and the mean was taken for each ant. The mean maximum stability margin did not differ between ants that carried short and long fragments (1.97±0.12 mm, mean±s.d.; Student’s *t*-test: *t* = −1.10, *df* = 31, *p* = 0.28) and was therefore not further considered.

Changes in stability during stance phase were determined by comparing the stability margin of the supporting tripod in all anterior (*AEP*) and posterior (*PEP*) extreme positions of the legs. For every ant, the mean stability margin was calculated for both positions.

To investigate whether the ants require additional legs in ground contact to achieve static stability, we measured stability margins for 1) only the supporting tripod, 2) the *polygon* of all legs in stance phase (without dragging hind legs) and 2) the polygon of all legs in ground contact (including dragging hind legs). Whether a foot was lifted off the ground or dragged during protraction could be identified based on the position of the shadows in both the top view and side view recordings. The minimum stability margin during three full strides was determined for all three conditions (tripod, polygon, polygon+dragging) for each ant. For both types of fragments, stability was assumed to be achieved when stability margins were equal or greater than 0. To investigate whether stability is reduced in ants that carried long fragments, minimum stability margins were compared between short and long fragments.

Since workers with short and long fragments carried their fragments at different angles (i.e. the angle between the fragment and the surface, short: 38.6±11.2°, long: 49.0±7.3°), we investigated whether this change in the fragment angle was required to maintain static stability. We therefore calculated stability margins for ants with short and long fragments for an assumed range of fragment angles (0°–90°). From the difference between the mean measured and the simulated fragment angle, the two-dimensional position of *CoM*_t_ was calculated trigonometrically for every frame. The minimum stability margin for the adjusted position of *CoM*_t_ for each ant and fragment angle was determined for each of the three conditions, as described above.

We tested whether ants carrying a long fragment would also have been stable when carrying a short fragment with the same gait, and whether ants carrying a short fragment would have been stable when carrying a long fragment. Minimum stability margins for the adjusted *CoM*_t_ were determined as described above for each ant, angle and condition (tripod, polygon, polygon+dragging).

### Excursion of the Neck Joint

To investigate whether the adjustment of the fragment’s position is limited by the ants’ physiological ability to move their heads up and down, positional changes during simulated head tilt and the required force were determined. Individual ants (body mass range: 2.62–8.98 mg, *n* = 11) were killed by exposing them to chloroform vapor. Subsequently, the ant’s legs were removed at the coxa-trochanter joint, so that the ant could be mounted laterally onto a glass plate with the head and at least half of the first thoracic segment exposed. The gaster and the posterior dorsal part of the thorax were fixed to the glass plate with dental cement (ESPE Protemp II, 3 M). A micromanipulator was used to slowly move (0.15±0.06 mm s^−1^) the ant against a U-shaped fixed tungsten beam (spring constant: 18.04 N m^−1^), so that the head was forced to tilt. The ant’s head and thorax positions and the displacement of the end of the beam were recorded from above during the whole movement with a video camera at 15 Hz (Basler A602f, Basler Vision Technologies, Ahrensburg, Germany) mounted on a stereomicroscope.

The head-thorax angle ε was digitized in every frame using two reference points on the ant’s thorax (posterior end of the thorax and base of the anterior mesotonal spines) and head (mandible tip and occipital spine). The angle at which the laterocervical plate (including the base of the forelegs’ coxae) started to be pulled forwards and soft tissue became visible was measured for each ant.

All positions were corrected for the movement of the glass plate. The pivot (i.e. the exact position of the neck joint) was determined by fitting a circle to the mandibles’ positions using a least radial square method. The torque was calculated for every frame, based on the displacement of the tungsten beam and hence the force *F*, the length of the lever arm *r* (i.e. the distance between the tip of the beam and the pivot) and the angle between the force vector *F* and the lever *r*.

Since we found that the absolute torque depended on the original position of the head, which varied in different ants, we calculated the change in torque relative to a head-thorax angle ε of 135°, which represented the minimum angle measured in all tested ants.

### Statistics

Data were tested for normal distribution and homogeneity of variances. *ANOVA* and *t*-tests were conducted for normal distribution and homogeneous variances, Welch *t*-tests for normally distributed data with heterogeneous variances, and Mann-Whitney U tests for non-normally distributed data.

## Results

### Gait Pattern

**Figure 2:**
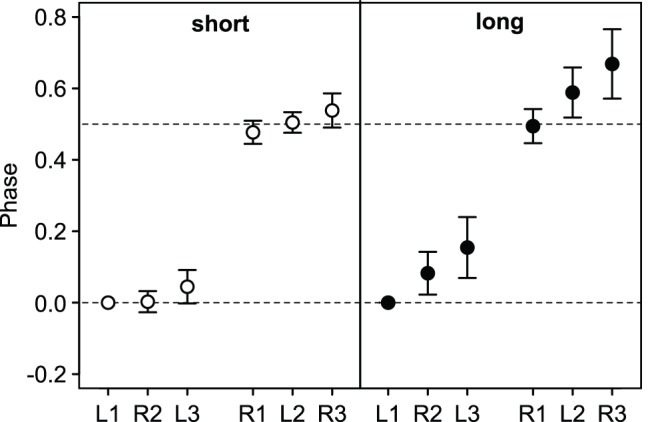
Phases (i.e. fraction of a full cycle) at which a leg was protracted after the left fore leg (L1) had lifted off the ground. Negative values indicate that the foot was protracted prior to L1. L, left and R, right body side; 1, 2 and 3 fore, mid and hind legs.

**Figure 3:**
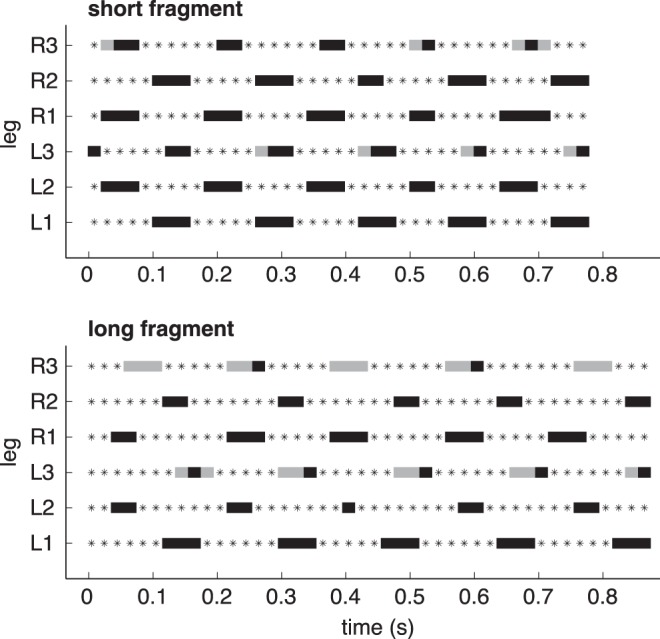
Typical gait patterns of ants carrying short and long fragments. Dotted lines indicate retraction and solid bars protraction. Black bars show legs off the ground, whereas grey bars indicate dragging. L, left and R, right body side; 1, 2 and 3 fore, mid and hind legs.

Ants carrying short and long fragments usually protracted their legs in the order L1, R2, L3, R1, L2, R3, L1, etc. (L and R, left and right body side; 1, 2, 3 fore, mid and hind legs, respectively, Fig. 2). All ants had at least three legs in ground contact at any given time. As typical for a tripod gait, the three legs of one tripod (L1-R2-L3 or R1-L2-R3) were protracted almost simultaneously. However, the delay in phase between the three legs of a tripod was significantly larger for ants carrying long fragments than for ants carrying short fragments (short vs. long: delay in phase between fore leg and contralateral mid leg: Welch test *t*_17.80_ = −4.67, *p*<0.001, between middle leg and contralateral hind leg: *t*_27.28_ = −2.32, *p*<0.05, Fig. 2). Because of the overlapping stance phases of legs from consecutive tripods, ants with short fragments were supported by a *polygon* of more than three legs in stance phase during 48.8±8.4% of a full stride and ants with long fragments for 74.8±13.2% of a full stride (Fig. 3). This difference was highly significant (*ANOVA, F*_1,31_ = 38.69, *p*<0.001).

Additionally, load-carrying ants did not always lift their hind legs off the ground during protraction but dragged them across the surface (Fig. 3). Mean absolute duration of protraction did not differ between ants with short and long fragments (short: 58.1±7.7 ms, long: 55.3±6.3 ms, *t*-test: *t*_31_ *= *1.12, *p* = 0.27). However, the relative duration of *dragging* during protraction was significantly higher in ants with long fragments (short: 38.8±13.5%, long: 67.6±12.8%, *t*-test: *t_31_* = −6.23, *p*<0.001).

### Stability Margin

**Figure 4:**
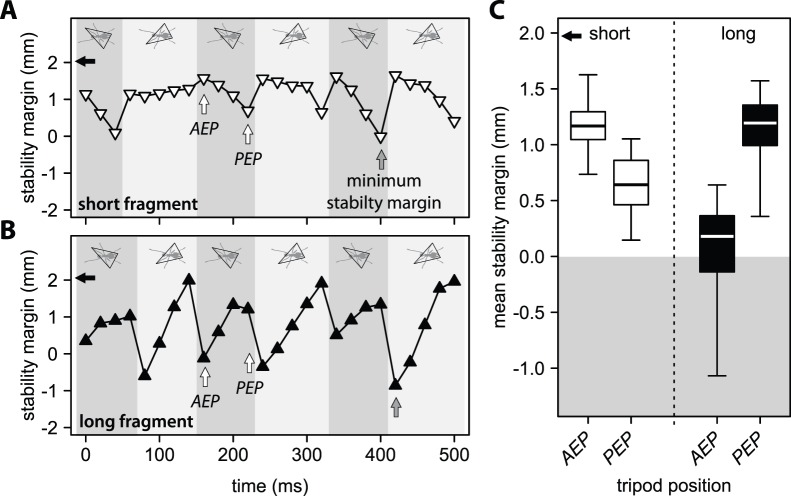
Variation of tripod stability margin during three full strides for an ant carrying A) a short and B) a long fragment. Diagrams at the top of the graphs illustrate the supporting tripod. White arrows indicate the anterior (*AEP*) and posterior (*PEP*) extreme positions of a tripod during one step. The minimum stability margin during the three full strides is indicated with a grey arrow. Black arrows show the maximum possible stability margin. C) Mean stability margin for tripods in their anterior and posterior extreme positions during three full strides for ants that carried short and long fragments. The black arrow indicates the mean maximum stability margin. Box plot shows medians (centre lines) and inter quartile ranges (boxes); whiskers indicate the highest and lowest values.

As the ant’s centre of mass moves forwards, the stability margin of the supporting tripod changes during stance phase. For ants with short fragments, stability margins were typically highest at the beginning of stance (*AEP*) and reached their minimum at the end of stance (*PEP*) when *CoM*_t_ was closest to the anterior boundary of the tripod (Fig. 4A). Mean stability margins were significantly lower in the legs’ posterior than in their anterior extreme position (Wilcoxon signed rank test: *V* = 165, *n* = 18, *p*<0.001, Fig. 4C). By contrast, for ants with long fragments, *CoM*_t_ was shifted posteriorly and stability margins were typically lowest at the beginning of stance (*AEP*) when *CoM*_t_ was closest to the posterior boundary of the tripod of support (Fig. 4B). Here, mean stability margins were significantly higher at the end of a stance (*PEP*) than at the beginning (*AEP,* Wilcoxon signed rank test: *V* = 0, *n* = 15, *p*<0.001, Fig. 4C). Thus, ants with short fragments would benefit from additional fore legs in ground contact, whereas additional hind legs would be advantageous for ants with long fragments. However, we found that for both short and long fragments, hind legs (including dragging ones) were added more frequently than front legs (Wilcoxon signed rank tests: short fragments: *V* = 0, *n* = 18, *p*<0.001, long fragments: *V* = 0, *n* = 15, *p*<0.001), suggesting that the ants did not add specific legs to achieve static stability.

**Figure 5:**
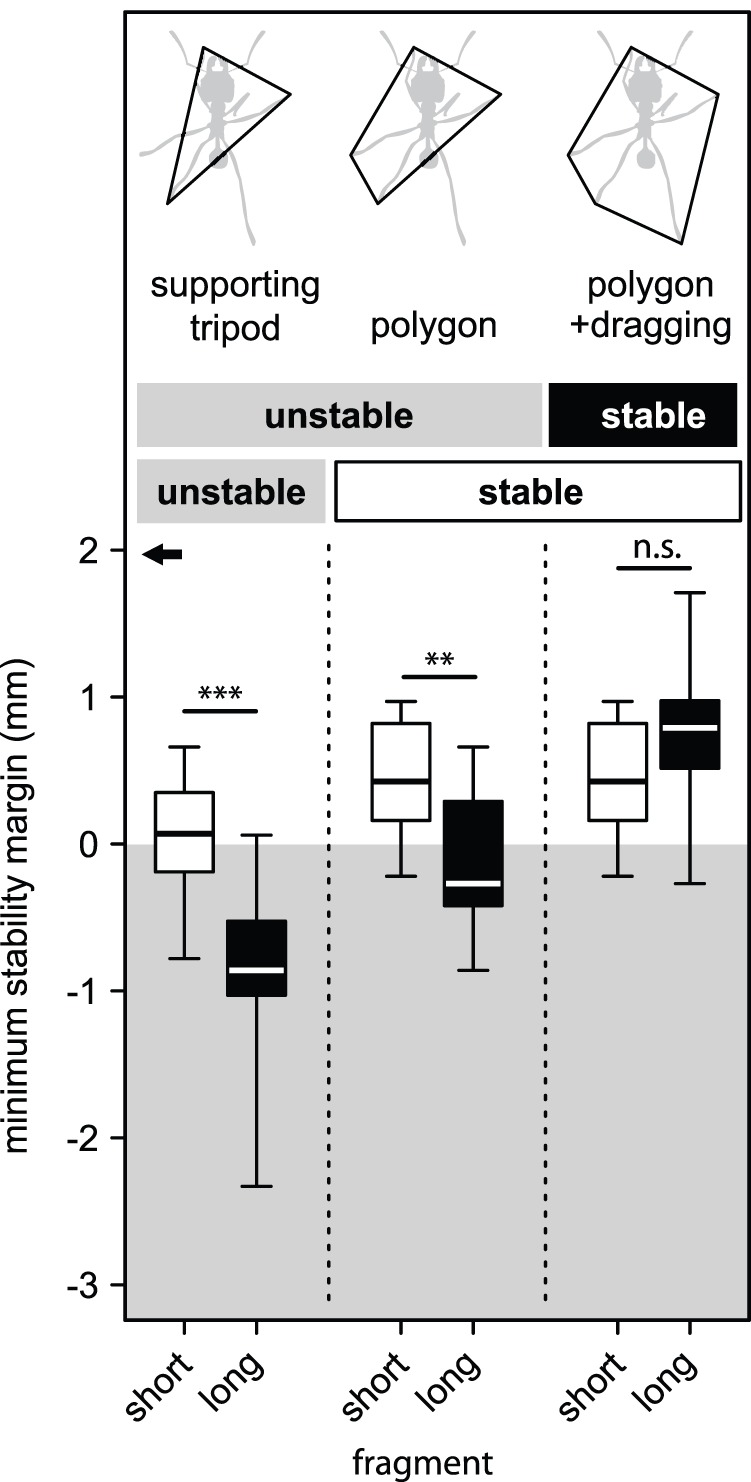
Minimum stability margin for ants that carried short and long fragments considering the supporting tripod only, the polygon of legs in stance and the polygon of all legs in ground contact (including the dragging hind legs). The black arrow indicates the mean maximum stability margin of a tripod. Box plot shows medians (centre lines) and inter quartile ranges (boxes); whiskers indicate the highest and lowest values. Asterisks demonstrate significance levels (**: *p*<0.01, ***: *p*<0.001).

Assuming that the ants walked with a non-overlapping *tripod* gait (without any further legs in ground contact), the minimum stability margins were negative for many ants with short fragments (8 out of 18) and for all but one ant with long fragments (14 out of 15), the difference being highly significant (Mann-Whitney U test: *W* = 253.5, *n* = 15, *m* = 18, *p*<0.001, Fig. 5). Thus, for both types of fragments, most workers would not be able to run statically stable if they used only a non-overlapping tripod gait.

The majority of workers with short fragments (15 of 18) achieved static stability with additional legs in stance (polygon), whereas this was still insufficient for most ants (10 of 15) with long fragments (Fig. 5). Minimum stability margins for the *polygon* of legs in stance were again significantly lower for ants with long fragments (Mann-Whitney U test: *W* = 224, *n* = 15, *m* = 18, *p*<0.001, Fig. 5). Most ants with long fragments achieved stability margins greater than 0 only when their *dragging* hind legs were considered (Fig. 5). When all legs in ground contact (polygon+dragging) were considered, minimum stability margins were similar in ants that carried short and long fragments (Mann-Whitney U test: *W* = 86.5, *n* = 15, *m* = 18, *p* = 0.08, Fig. 5). Nevertheless, *CoM*_t_ was found to be unsupported at least temporarily in 3 of 18 workers with a short fragment and in 1 of 15 with a long fragment, representing the ant that carried the long fragment at the steepest angle (65.4°). However, none of these ants fell over during the recording. In all these ants, *CoM*_t_ was located close to the anterior end of the supporting polygon (stability margin ≥ −0.27 mm) and was unsupported for no more than one frame (20 ms) until the fore leg from the next tripod touched the ground to re-support *CoM*_t_. Additionally, the fragment angle increased while *CoM*_t_ was unsupported, suggesting that the ants were falling forwards and took advantage of dynamic stability.

With a non-overlapping *tripod* gait only, most ants carrying short fragments would have been stable for fragment angles between 10° and 25°, which is lower than their actual mean fragment angle α of 38.6°. Thus, holding a short fragment at a smaller angle would have allowed the ants to run without additional legs in ground contact. By contrast, ants carrying long fragments with a non-overlapping tripod gait would not have been stable for any fragment angle between 0° and 90°.

**Figure 6:**
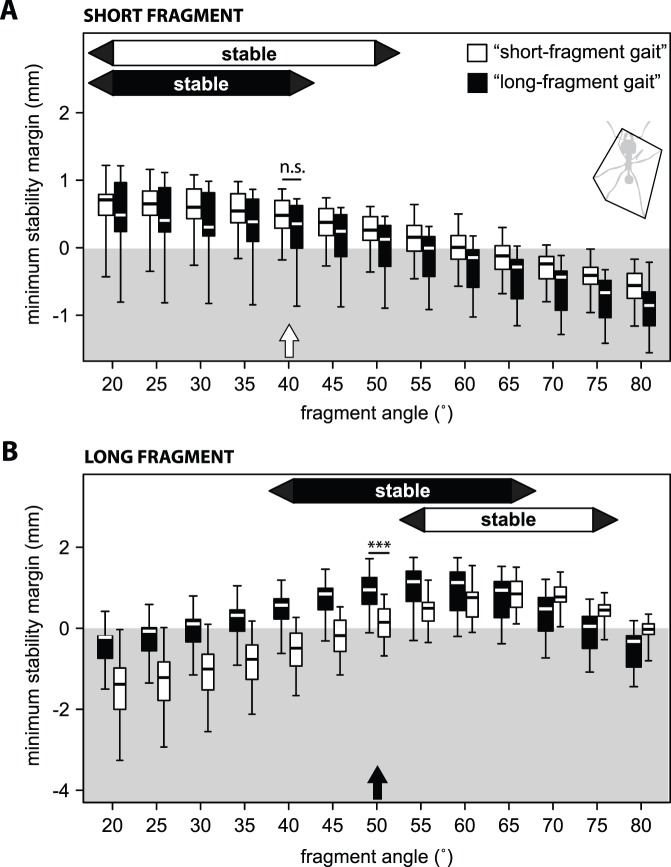
Stability margins of ants carrying A) short and B) long fragments calculated for different fragment angles. The white box plots show the result for ants assumed to move with the gait of ants with short fragments; black box plots show the result for ants assumed to move with a “long fragment gait”. The white arrow indicates the actual mean fragment angle of ants that carried short fragments and the black arrow the one for ants with long fragments. Box plot shows medians (centre lines) and inter quartile ranges (boxes); whiskers indicate the highest and lowest values. Asterisks indicate significance levels (***: *p*<0.001).

Taking into account all legs in ground contact, the majority of ants with short fragments would have been able to run statically stable with fragment angles between 0° and 50° (Fig. 6A), which includes the range of their actual mean fragment angles (23.2–48.8°, with two exceptions >50°, *n* = 18). Short fragments could also have been carried with the gait of workers with long fragments, yet this would have slightly narrowed the range of possible fragment angles to <45° (Fig. 6A). However, at the actual fragment angle of short fragments (∼40°), no significant difference in stability between the gait of ants with short and long fragments was found. Thus, the observed differences in gait pattern between ants with short and long fragments do not serve to support the transport of short fragments.

By using additional legs in ground contact (polygon+dragging), most ants carrying long fragments would have been stable for fragment angles between 40° and 65° (Fig. 6B). In almost perfect agreement, the fragment angles of ants with long fragments ranged from 42.8° to 65.4° (with one exception: 30.9°, *n = *15). Ants carrying long fragments, but moving with the gait of ants with short fragments would have achieved static stability for fragment angles between 55° and 75°. Thus, ants would have been able to maintain the gait used for short fragments by adjusting the fragment angle to a minimum of 55°. At the actual fragment angle of long fragments (∼50°), stability margins were significantly higher for the gait of ants with long fragments than for the gait of ants with short fragments (Mann-Whitney U test: *W* = 118, *n* = 15, *m* = 18, *p*<0.001, Fig. 6B), showing that the ants’ gait adjustments improve stability during the transport of long fragments.

### Estimation of Fragment Angle Range from the Excursion of the Neck Joint

**Figure 7:**
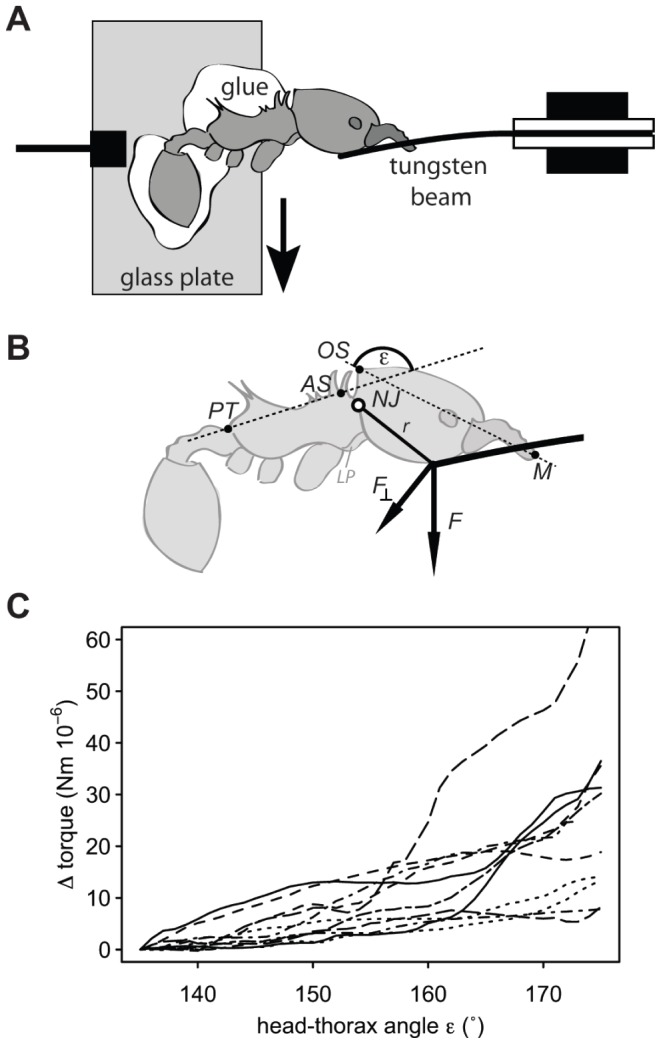
Experimental setup (A,B) to measure the ants’ maximum range of the neck joint and C) the absolute change in torque acting on the neck joint (*NJ*) with increasing head-thorax angle ε. A) Individual ants glued laterally onto a glass plate were moved with a micromanipulator against a U-shaped tungsten beam, forcing the head to tilt. B) The torque acting on the neck joint (*NJ*) was calculated based on the displacement of the beam and therewith the force *F*, the length of the lever *r* and the angle between the force vector *F* and the lever *r* (*PT* posterior end of the thorax, *AS* anterior mesonotal spine, *OS* occipital spine, *M* mandibles’ tip). C) Individual lines show different ants.

During the simulated head tilt, the laterocervical plate started to be pulled forwards and soft tissue became visible for head-thorax angles ε between 137° and 163° (155±8°, n = 11 ants). We never observed such a displacement of the laterocervical plate in naturally load-carrying ants. For many of the measured ants, this range of the head-thorax angles coincided with a strong increase in the torque acting on the neck joint (Fig. 7C), suggesting that the neck joint had reached its maximum excursion, where the head presses against the thorax and starts to displace the laterocervical plate. Thus, our results suggest that the ant’s ability to raise their heads is limited physiologically to a head-thorax angle of approximately 155°. Since grass-cutting ants mainly adjust their fragments’ position by moving their heads up and down, and as a head-thorax angle of 155° approximately corresponds to a fragment angle of 30° [19], the ants’ inability to raise their heads further likely constrains fragment angles to values >30°.

Our setup did not allow measuring the *lower* physiological limit of the excursion of the neck joint since the glass plate limited the head movement in this range. Nevertheless, our measurements clearly showed that neck angles can be significantly smaller than a head-thorax angle of 95° required to carry fragments vertically (90°). Thus, the *maximum* fragment angle is not limited by the ants’ ability to move their heads.

## Discussion

This study demonstrates that load-carrying grass-cutting ants run close to their limit of stability. Static stability was only achieved when the ants’ supporting tripod was further stabilized by additional legs in ground contact. For ants with short fragments, an additional fore leg in stance was mostly sufficient to maintain static stability. For ants with long fragments, this was still insufficient and they only achieved stability by dragging their hind legs, i.e. keeping them in ground contact during protraction. We found that the ants maintained stability both by adjusting their gait pattern and the angle of the fragment. Additional legs in ground contact during the transport of long fragments likely limit the ants’ running speed [10]. In fact, in previous studies running speed was found to be reduced by 20–40% in ants with long fragments compared to short fragments of the same mass, which also resulted in a lower transport rates [20], [23].

For ants with short fragments, minimum stability margins occurred near the end of a step, when the centre of mass was near the anterior edge of the tripod. This is similar to the situation described for running cockroaches [11]. By contrast, ants that carried long fragments have their centre of mass shifted backwards. In these ants, minimum stability occurred at the beginning of each step, when the centre of mass was near the posterior margin of the supporting tripod. These results explain our previous finding that ants with short fragments usually fall forwards or to the side, whereas ants with long fragments mainly fall backwards [19].

Although most carrying ants in this study exhibited continuous static stability, we did observe a few cases where the centre of mass was outside the polygon of supporting legs but the ants did not fall over. Static instability in insects was previously observed in fast-running ants and cockroaches that exhibit phases with fewer than three legs in ground contact [11], [24]. These insects usually “fall” forwards only briefly until new supporting legs contact the ground, a condition termed “dynamic stability”. Animals can be dynamically stable when the leg cycling time is less than the time taken for the body to fall (the latter time usually scales with the square root of hip height). We observed dynamic stability mainly during the transport of short fragments, where the centre of mass was mostly shifted forwards. This may explain why ants with long fragments are more likely to fall over than ants with short fragments [19], even though they exhibit similar stability margins (Fig. 5).

Our results show that theoretically, workers would have been able to maintain sufficient stability without changing their gait, only by adjusting the fragment angle. If ants had carried short fragments at a lower angle (between 10° and 25°), they would have been able to run with a non-overlapping tripod gait, which would have allowed them to run faster. Similarly, had the ants carried long fragments at a steeper angle (>55°), they would have achieved static stability with the gait of ants carrying short fragments (i.e. with fewer legs in ground contact and a higher running speed). Why are the ants not using these seemingly better fragment angles? Grass-cutting ants adjust the fragment position by moving their head up and down at the neck joint [19]. The ants’ ability to raise their heads to achieve smaller fragment angles is likely to be limited physiologically by the working range of the neck joint. Our results suggest that the minimum fragment angle is ca. 30°, larger than required for optimal stability. Smaller fragment angles cannot be achieved by further reducing the angle between the head and the fragment, because the mandibles are already almost parallel to the edge of the fragment, and likely near the limit where they lose the ability to firmly grasp the fragment. By contrast, maximum fragment angles are not limited by the ants’ neck joint. However, workers may avoid carrying fragments at a steeper angle to prevent the lower end of the fragment from touching the ground. Grass-cutting ants do not hold their fragments at the very end, but slightly further away from it. For mean fragment angles greater than 60°, the lower end of the fragment can indeed touch the ground, thereby providing an upper limit for the fragment angle (see Supporting Information S1). Additionally, the ants’ fragment angle typically changes during running. A fragment angle of 49° just ensures that the fragment does not touch the ground during a run (see Supporting Information S1). These estimates apply to flat surfaces, and the problem would get worse on uneven ground. Any further increase of the fragment angle could only be achieved by raising the body off the ground or by reducing the variation of the fragment angle during running.

The ants’ optimal fragment positions and gait patterns may also depend on a number of environmental conditions such as trail gradients, changes in wind conditions, and obstacles that interfere with the fragment. Running speeds in leaf-cutting ants have been shown to depend on such environmental conditions (trail gradients [25], [26], height constraints [27]), and it is likely that these effects are based on the ants’ need to maintain stability. We previously demonstrated that grass-cutting ants can maintain stability by adjusting the fragment angle by head movements on trails with inclines (+20°) or declines (−20°), but this re-orientation did not fully compensate the predicted shift of the centre of mass [19]. On even steeper slopes, maintaining static stability will become impossible [28], [29] and ants may only be able to avoid falling by clinging to the surface using claws or adhesive structures. Consistently, many leaf-cutting ants foraging on trees have been observed to drop fragments to the ground, probably to reduce the torque tending to detach their feet from vertical tree trunks [30], [31].

The need to have a larger number of legs in ground contact will likely reduce the ants’ running speed [32], [33] and thus the rate at which leaves are transported to the nest, but it may also increase the energetic cost of transport for longer fragments as a result of the lower running speed [23]. Hence, to maximize transport rate and minimize cost of transport, workers should choose shorter fragments that interfere less with their locomotion. In fact, grass-cutting ants show a tendency to avoid the transport of long fragments. Given a choice of long and short fragments, grass-cutting ants prefer to pick up shorter ones, independent of their mass [20]. The ants often cut fragments into smaller pieces instead of transporting them; the probability for this behavior increases with fragment length [34], [35]. However, under natural conditions ants carry fragments that are longer than their preference for picking them up [18], [20]. This discrepancy may be explained by the high metabolic cost of cutting: longer grass fragments yield more material per unit cutting effort, because the length of the cut (i.e. the width of the grass blade) remains invariant, irrespective of fragment size [20], [36]. Thus, the length of the carried fragments may represent a trade-off between minimizing both the costs of cutting and transport, and maximizing the delivery rate of material.

Biomechanical constraints not only influence the ants’ transport performance but also the ants’ ability to lift fragments from the ground. This may have a direct influence on the (active or passive) selection of fragments. Load size selection is a critical process that ultimately determines the foraging success of a leaf-cutting ant colony. It depends on a number of ecological factors, including food quality and availability [14], [37], foraging distance [38] as well as daytime, temperature and foraging history [39]. As leaf-cutting ants usually do not carry their fragment straight to their nest but form transport chains [30], [34], [40], workers often have to lift fragments from the ground and bring them into their carrying position. Lifting inevitably gives rise to large torques that the ants must overcome. It is unknown whether or to what extent load selection in leaf-cutting ants while collecting dropped fragments is mechanically determined by the ants’ ability to lift fragments from the ground.

Our results underline that maintaining stability is crucial for the load transport in leaf-cutting ants. Analyzing the biomechanical constraints that influence the cutting, handling and carrying of fragments will be essential for the understanding of this complex foraging system.

## Supporting Information

Figure S1 **Calculation of the fragment angle α_1_ for which the lower end of the fragment touches the ground neck joint (** ***NJ*** **).** *f*: height of the lower end of the fragment, *h*: height of the mandibles above the ground, *l*: length of the head (tip of mandibles – posterior end of head), *d*: distance between the lower end of the fragment and the mandibles, γ: head angle, *NJ* neck joint. Indices 0 and 1 indicate prior and after angle change.
(EPS)

Supporting Information S1 **Estimation of the angle at which the fragment would touch the ground.**
(DOC)
