# Supplementary material for: How Load-Carrying Ants Avoid Falling Over: Mechanical Stability during Foraging in Atta vollenweideri Grass-Cutting Ants
Source: PLoS One. 2013 Jan 2;8(1):e52816. doi: 10.1371/journal.pone.0052816 (PMC3534694; doi:10.1371/journal.pone.0052816)
Supplement: Supporting Information S1 — Estimation of the angle at which the fragment would touch the ground. (DOC) [file pone.0052816.s002.doc]

**Supporting Information S1**

*Estimation of the angle at which the fragment would touch the ground*

Ants held long paper fragments 1.84±0.40 mm away from the lower end of the fragment (mean distance along the fragment between its lower end and the ant’s mandible tip, *n*=15, Fig. S1). Any change of the fragment angle α is linked to a change in the mandibles' height *h* and consequently in the height of the fragment *f* above the ground (Fig. S1). Assuming that the height of the neck joint remains constant, the height *f*_1_ of a fragment above the (flat) ground after a change of the fragment angle from α_0_ to α_1_ can be estimated as

$f_{1}=h_{0}-d \sin\alpha_{1}-l \left[\sin\left(\alpha_{1}-\alpha_{0}+\gamma_{0}\right)-\sin\gamma_{0}\right]$ (1)

where *h* is the height of the mandibles, *l* the length of the head (from the tip of the mandibles to the posterior end of the head), γ the angle of the head relative to the surface and *d* the distance between the mandibles and the lower end of the fragment (Fig. S1). The height *h*_0_ of the mandibles above the ground for the ants that carried long fragments at a mean angle α_0_ of 49.0˚ was 2.17±0.40 mm (measured from the video recordings). The head length *l* measured for a range of workers with a mean mass of 4.50 mg averaged 3.22 mm. The head angle γ for ants carrying long fragments was 18.8˚ (angle between the surface and the line between the occipital spine at the back of the head and the mandibles' tip, [19]). This angle does not exactly represent the angle between the surface and the line through the mandible's tip and the pivot point, but it is slightly higher, leading to a conservative estimate. Based on these parameters, the height of a fragment *f*_1_ above the ground after an angle change can be calculated as

$f_{1}=2.17-1.84 \sin\alpha_{1}-3.22 \left[\sin\left(\alpha_{1}-30.2^{\circ}\right)-\sin\left(18.8^{\circ}\right)\right]$ (in mm) (2)

Hence, *f*_1_ is smaller than 0 for mean fragment angles α_1_ greater than 60˚, showing that the lower end of the fragment can indeed touch the ground. Additionally, the ants' fragment angle typically changes during running (maximum deviation from the mean fragment angle α during the transport of long fragments: 8.2˚±2.2˚, *n*=15). Thus, a fragment angle α of 49˚ just ensures that the fragment does not touch a flat surface during a run. If the ants walk on uneven ground, fragments might touch the surface for even smaller angles.
